# Parotid Masson's Tumor in a 29-years-old woman: A Case Report

**DOI:** 10.22038/IJORL.2023.58567.3288

**Published:** 2023-05

**Authors:** Saleh Mohebbi, Shahriar Zohourian Shahzadi, Ali Jamshidi Naeini

**Affiliations:** 1 *ENT and Head & Neck Research Center, Five Sense Health Institute, Iran University of Medical Sciences, Tehran, Iran.*; 2 *Department of Pathology, Iran University of Medical Sciences, Erfan Hospital, Tehran, Iran.*

**Keywords:** Intravascular papillary endothelial hyperplasia, Masson's tumor, Parotid gland, Case Report

## Abstract

**Introduction:**

Intravascular papillary endothelial hyperplasia (IPEH) is a papillary hyperplasia of the endothelial vascular cells, also called Masson's tumor. Masson's etiology and risk factors remain unclear but trauma and vascular pathologic conditions may start the tumor process from its common regions like extremities. Common presentations include swelling and mild pain. Our Radiologic modality of choice is Contrast-enhanced MRI which can help us before operating parotidectomy, the gold standard of tumor treatment. As presented in this study, Parotid Masson's tumor, is a very rare form of Masson's,making it even more exceptional.

**Case Report:**

This paper reports a case of a 29-years-old woman with a mass in herright parotid gland from 17 years ago, which has slowly increased in size during these years. She underwent a total parotidectomy following unsuccessful Fibrovein injections, which caused her inflammation. Embolization was performed before the resection to decrease the risk of its hemorrhage. Postoperative follow-up confirmed the reliability of this treatment method as the patient declared no side effects. Apart from its tough diagnosis, since Masson's tumors, especially the ones that emerge in the parotid, are rare, we decided to introduce this case to deliver more information about the treatment and diagnosis of this rare disease to other colleagues.

**Conclusions:**

The prognosis of parotid Masson's is admirable following a total resection. The patient had no postoperative complaints with no need for multiple visits after resection.

## Introduction

Intravascular papillary endothelial hyperplasia (IPEH), also known as Masson's tumor, was first presented by Pierre Masson in 1923. 

It is also called vegetative intravascular hemangioendothelioma as its similarity to angiosarcoma (1). It is papillary hyperplasia of endothelial vascular cells, which frequently develops in medium-sized veins (2).

Masson's tumor etiology is indistinct; and is a multifactorial disease. Local trauma and vascular settings such as hemangiomas, blood stasis, and pyogenic granulomas can act as triggers for the tumor; however, triggers and stimulating factors still stand unclear in almost 70% of patients (1). 

Masson hemangioma is not a true neoplasm. It composes the organization and recanalization of thrombi within vessels, causing the formation of papillary architectures with occasional plump endothelial cells and occasional anastomosing channels within thrombi. The constant finding is no evidence of bizarre endothelial cells, prominent mitosis, or necrosis in the sections. IPEH generally emerges on the skin region and subcutis, containing about 2% of vascular tumors in these areas; however, all head, neck, trunk, and extremities incline this tumor. Histopathology has a key role in IPEH's diagnosis (3). 

IPEH's most common presentations are swelling gland enlargement and mild pain. Contrast-enhanced magnetic resonance imaging (MRI) can help in preoperative planning; also, total or subtotal parotidectomy and extracapsular dissection of the lesion are some of the practical therapeutic interventions, so surgical treatment is our choice (1,3).

## Case Report

This literature adhered to all ethical principles and was approved by the Iran University of Medical Sciences Ethics Committee. Concerning privacy, all the steps of this study and its procedures were fully described. In the end, consent was obtained from the patient regarding this publication.

The case we want to present is a 29-years-old woman complaining a problem with her right Parotid. In the primary examination, swelling and enlargement in the right parotid were obvious; However, she did not complain about other symptoms like facial movement difficulties, hypogeusia, dysphagia, and mastication impairment. Also, no signs of neurological deficits or paralysis were found.

The oropharyngeal examination had no positive signs as well. The patient said that she has been aware of the discomfort since she was about 12, but it did not cause her any severe symptoms or pain, therefore during these years, she made several visits to the doctors, also Fibrovein (Sodium Tetradecyl Sulfate) was injected to the mass twice. The inflammation did not get better, but it also got worse.

Contrast-enhanced magnetic resonance imaging showed a hypervascularized lobulated mass of approximately 2.5cm in diameter within the superficial lobe of the parotid gland. Also internal bleeding in the lesion was found. Finally, the patient became a candidate for surgical resection.

**Fig 1:**
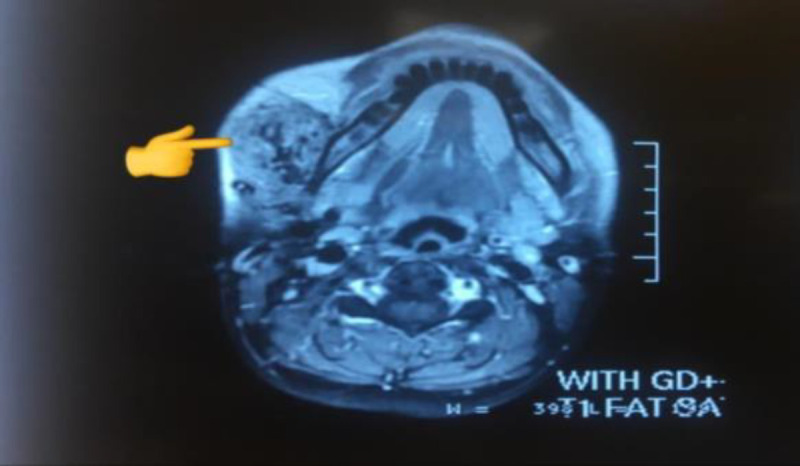
T1-weighted FAT-SAT axial sequence showing an irregular enhancement following the medium contrast uptake (Pointer)

**Fig 2:**
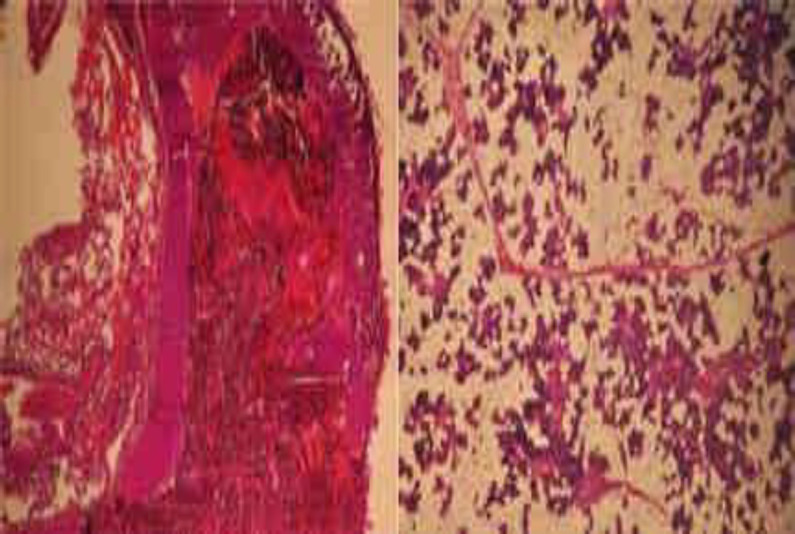
Intravascular papillary proliferation of endothelial cells (Left) and an organizing thrombus (Right)

An embolization procedure suggested a vascular mass presence and led to the shrinkage of the mass. The patient underwent a Total parotidectomy with the facial nerve preserved, and the specimen was sent to the pathology laboratory. The specimen was sent in formalin in two separate containers and consisted of:1-Multiple fragments of irregular brownish soft tissue measuring 2.6 x 2.4 x 1.4 cm and 2-One fragment of irregular grayish-creamy soft to rubbery tissue measuring 3.3 x 2.8 x 1.2 cm. T1-weighted FAT-SAT axial sequence shows evidence of a tumor in the parotid (Figure 1). Microscopic diagnosis suggested a Deep parotid vascular mass (Masson's hemangioma). Moreover, the gland was free from a tumor (Figure 2). No signs of necrosis and mitoses were perceived. Postoperative follow-up and visits proved the resection was successful, and the patient did not mention any adverse effects or complaints after the operation.

## Discussion

Masson's tumors are divided into three types: the most frequent one is a pure one which arises in dilated vascular spaces.

Hemangiomas, pyogenic granuloma, and vascular malformations form the secondary mixed type and an extravascular hematoma forms the latter type (4). 

Clinically Masson's tumor resembles benign lesions and malignant neoplasms on ultrasound, and main differential diagnoses include hemangioma, Kaposi sarcoma, and pyogenic granuloma. The most common manifestation of this tumor is a solid and tender slow-growing mass in the lateral region of the head and neck, but other regions are also possible to be involved, such as the sino-nasal tract (5,6).

Many authorities and researchers have already established endothelial cell proliferation as a main part of the Masson's pathogenesis. This occasion leads to vessel obstruction and red infarction (3). Following the thrombus formed in the vessel, macrophages release bFGF (basic fibroblast growth factor) to the point where some studies identify IPEH as a rare form of thrombus assorted with fragmentation (7,8). 

The age range involved in this tumor is widespread, and there is no sex predilection, which means men and women are equally affected (2), although Chang in 2012 declared that women are more at risk due to high levels of progesterone and estrogens (9). 

In the initial stages of lesion development, ferritin positivity, vimentin, and factor VIII-related antigen positivity has been detected in the final ones (10, 11). In this case, no signs of declared objectives were spotted. MRI Is the best radiologic modality for Masson's diagnosis. In this case, post-contrast T1-weighted images confirmed heterogeneous hyperintensity and most cases previously reported in other studies. Finally, Hyper vascularized lesion was considered following the T2 heterogeneous intensity and irregular medium contrast uptake in T1 weighted FAT-SAT sequences.

Total and sub-total parotidectomy is our main choice of resection and the gold standard surgical technique. In our case, one of the vital challenges of surgical treatment of the lesion was lesion inflammation which increases the risk of bleeding. The other one was a need for a fine surgery to prevent the facial nerve from any damage that can cause severe deficits like paralysis (floppy cheek and droopy mouth).

According to the pathology report of this case, deep parotid vascular mass was detected, which facilitated the diagnosis of Masson's hemangioma (2.6 x 2.4 x 1.4 cm). There were no signs of necrosis or any other pathological feature.

## Conclusion

What is unique about this tumor, especially this case, is the possibility of mimicking other neoplasms like hemangioma and Kaposi sarcoma, so it can be misunderstood and may lead us to the wrong approach. Besides, we do not expect Masson's in the parotid gland. 

Many parotid Masson's respond well to parotidectomy based on the results obtained from this case. However, we invite the other colleagues and specialists to do more research about this title and keep these rare forms of Masson's cases in mind as we may face a rare tumor formed in a region sporadically.
